# Large datasets of single carbon and glass fibre mechanical properties obtained with automated testing equipment

**DOI:** 10.1016/j.dib.2021.107085

**Published:** 2021-05-01

**Authors:** Francisco Mesquita, Steve Bucknell, Yann Leray, Stepan V. Lomov, Yentl Swolfs

**Affiliations:** a Department of Materials Engineering, KU Leuven, Kasteelpark Arenberg 44 box 2450, 3001 Leuven, Belgium; b Dia-Stron Ltd., 9 Focus Way, Andover SP10 5NY, United Kingdom

**Keywords:** Fibres, Strength, Elasticity, Mechanical testing

## Abstract

This data article presents fibre mechanical properties acquired using automated single fibre tensile testing equipment. The raw data consists of the fibre diameter, gauge length and load-displacement diagrams. A total of 690 fibres were tested across four carbon and one glass fibre type. The largest dataset, for the T700S carbon fibre, consists of 217 data points. After performing a compliance calibration, the load-displacement diagrams are converted into stress-strain diagrams from which the tensile modulus and strength are extracted. The data presented in this article can be used as input data for models or for data processing during determination of other properties experimentally. The interpretation of the data can be found in [1]. The data is hosted in the *Mendeley Data* repository at [2].

## Specifications Table

**Table:** 

Subject	Materials science
Specific subject area	Fibre reinforcements for high performance lightweight materials
Type of data	Table (comma separated values)
How data were acquired	LDS0200 laser diffraction system, LEX 820 tensile testing machine
Data format	Raw (fibre diameter, gauge length), filtered (force-displacement diagrams) and analysed (stress-strain diagrams, tensile modulus, tensile modulus variation with strain and strength). The initial data points in some force-displacement diagrams have been removed when the load did not increase immediately.
Parameters for data collection	Fibres were extracted from a dry bundle. Only fibres that had a length higher than the gauge length were considered. Fibres for which the diameter was not measured were not considered for the study. The tensile modulus was not calculated for fibres that presented drops in the force-displacement diagram.
Description of data collection	The automated testing equipment used a laser diffraction system to measure the fibre diameter and a tensile tester to record the force and displacement. The gauge length was determined by the intersection of the force-displacement diagram with the displacement axis.
Data source location	Institution: KU Leuven City: Leuven Country: Belgium Latitude and longitude for collected data: 50.860481, 4.683385
Data accessibility	https://data.mendeley.com/datasets/ygyym4vy6b/1 at [2].
Related research article	F. Mesquita, S. Bucknell, Y. Leray, S. V. Lomov, Y. Swolfs, Single carbon and glass fibre properties characterised using large data sets obtained through automated single fibre tensile testing, Compos. - Part A Appl. Sci. Manuf. 145 (2021). https://doi.org/10.1016/j.compositesa.2021.106389

## Value of the Data

• Fibres are the main load carrying component in composite materials. Knowledge of their individual properties is highly important to understand the longitudinal tensile failure of composite materials. The data acquired can be used to improve the reliability of modelling studies and to determine other composite material properties that require the knowledge of fibre properties. The data was used, for example, to perform a benchmarking exercise between several models that predict the longitudinal tensile strength of composite materials. Without a proper data set that could be compared with experimental data, all conclusions of such exercise would not be supported.
• Researchers who study composite material properties that are influenced by the tensile properties of fibres can benefit from this data set. For example, researchers characterising the tensile strength of unidirectional composites can learn how the composite material failure strain differs from that of the single fibres.
• The data can used to model the mechanical properties of composite materials, to validate other methods of fibre property acquisition or to ensure that the setup of another experiment is appropriate/accurate for the fibres used, based on their tensile strength and stiffness. Another researcher can also analyse the raw data to perform other statistical analysis that were not shown in the related research paper [1], namely by fitting a different type of probability distribution to the fibre strength.

## 1 Data Description

The file names start with the respective fibre type name: T700, 34–700, T300, HS40 and HYBON-2026. Three types of ASCII files are provided for each fibre type.

The first type of file, name ending with “analysed_data”, contains 8 columns. Each row of this file corresponds to a tested fibre. The data in the columns is the following:

• Column 1: Specimen index;
• Column 2: Fibre diameter, in µm;
• Column 3: Gauge length, in mm. This is the gauge length measured by the tensile testing equipment, corresponding to the gauge length for which the fibre supported 0.01 N;
• Column 4: Boolean indicating the presence of manually identified load drops in the force-displacement diagram. The Boolean takes the value 1 if the force-displacement presented such load drops;
• Column 5: Fibre strength, in MPa. This corresponds to the maximum force recorded in the force-displacement diagram, divided by the cross-sectional area;
• Column 6: Tensile modulus, in GPa. The tensile modulus was obtained from the stress-strain diagram for in a strain range from 0.1 to 0.6% for all fibre types except for the HS40 carbon fibre, for which it was measured between 0.1 and 0.3% strain. The strain ranges were chosen according to the BS ISO 11,566 standard [3]. The tensile modulus was only measured in fibres that did not show visible stress drops in the respective strain range;
• Column 7 and 8: Stiffening rate, in GPa per 1% of applied strain and Initial tensile modulus, in GPa. The two columns are presented together here due to their inherent relationship. A stiffness-strain diagram is obtained by determining the slope of the stress-strain diagram as a function of the applied strain. Each point in the stiffness-strain diagram corresponds to the slope of the stress-strain in a range of 0.2% of strain. The average strain was increased by 0.01% to obtain the following data point. A linear function was then fitted to the stiffness-strain diagram. The slope of that linear function corresponds to the stiffening rate and the intersection with the Y axis to the initial tensile modulus. This analysis was not performed if the force-displacement diagram showed visible load drops.

**Fig. 1:**
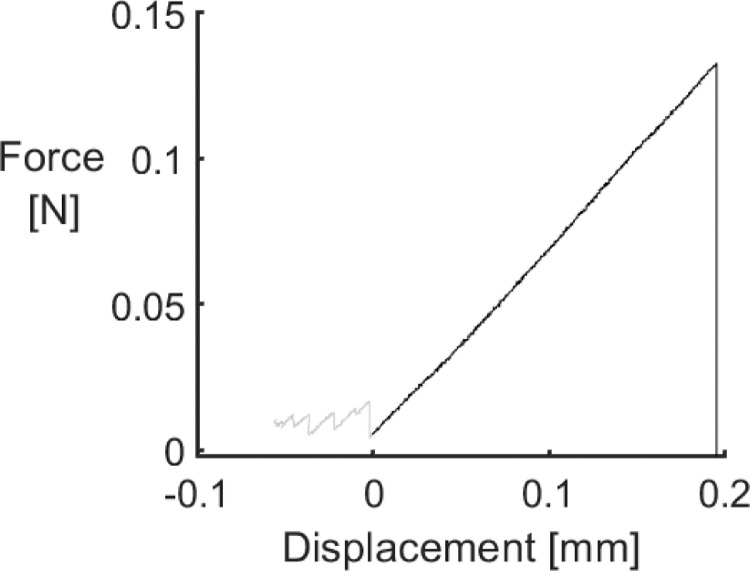
Example of a force-displacement diagram for a T700 carbon fibre where the force did not increase at the beginning of the test. The black line corresponds to the filtered data.

The second type of file, name ending with “force_displacement”, provides the filtered force-displacement diagrams for each tested fibre. The only modification done to the raw data was the exclusion of the initial data points in the event that the force did not increase at start of the test. An example of such force-displacement diagram is shown in Fig. 1. The contains 3 columns:

• Column 1: Specimen index
• Column 2: Displacement, in millimetres;
• Column 3: Force, in N.

**Fig. 2:**
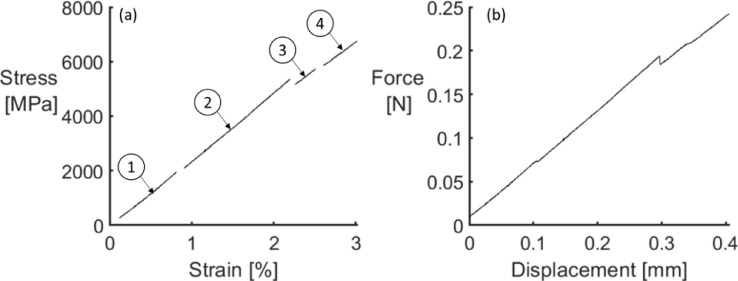
Example of a (a) stress-strain diagram for a T700 carbon fibre where stress drops were identified. The sections are referenced with the circled numbers. The corresponding force-displacement diagram is shown in (b).

The third type of file, name ending with “stress_strain”, provides the stress-strain diagrams for all tested fibres. The visible load drops were identified in the force-displacement diagrams and used to divide the stress-strain diagram into sections, as shown in Fig. 2. The file contains 4 columns:

• Column 1: Specimen index;
• Column 2: Strain, in%. The strain was determined after a compliance calibration following the method in ASTM C1557-14 [4];
• Column 3: Stress, in MPa. The stress was determined by dividing the force in the force-displacement diagrams by the respective fibre cross-sectional area;
• Column 4: Section index. For each fibre tested, the stress-strain diagram was divided into sections according to the presence of stress drops. In case a stress drop was identified, the following data points bellowing in another section. The number of sections in each stress-strain diagram depends on the number of stress-drops manually identified.

## 2 Experimental Design, Materials and Methods

### 2.1 Single fibres

Four different types of PAN-based carbon fibres and one type of E-glass fibre were analysed in this article. The nominal properties of each fibre type, specified by the manufacturer, and the number of fibres tested are shown in Table 1.

**Table 1:** Nominal properties of the fibres and number of tests performed.

Fibre type	Diameter [µm]	Tensile modulus [GPa]	Tensile strength [GPa]	Failure strain [%]	Number of tests
T300 (Toray)	7	230	3.53	1.5	105
T700S (Toray)	7	230	4.90	2.1	217
34–700 (Mitsubishi)	7	234	4.83	2.1	170
HS40 (Mitsubishi)	5	425	4.61	1.1	99
HYBON 2026 (PPG)	15	82	2.79	3.4	105

### 2.2 Automated single fibre testing

The single fibre tests were performed with the LEX/LDS automated testing equipment developed by Dia-Stron Ltd [5]. Each fibre was extracted from the bundle by hand with the assistance of a vacuum pen and then mounted onto two plastic tabs, where each tab holds one of the fibre ends. The bundle was separated into smaller bundles until only a few fibres were attached. This made it easier to obtain the tested fibres without breaking them.

The tabs were placed in a cassette with slots for 20 or 50 fibres. The cassette helps in obtaining an accurate gauge length by providing the slots for the plastic tabs with a pre-defined distance between them (12.24±0.10 mm in this work). Each tab had a V-shaped slit that helps aligning the fibre. The fibre should be long enough so that it does not only cover the gauge length but also the length of the tab. Each tab had a pocket for an adhesive where the fibre was fixed to the tab. A droplet of an ultraviolet (UV) curing adhesive was then placed over each tab to fix the fibre and tab. Each droplet of adhesive was cured individually by illuminating it with a UV lantern for 15 s. The entire fibre mounting procedure took 1–2 min per fibre.

When all the slots in the cassette have been filled, the cassette is mounted on the testing equipment. From this moment on, the procedure was automated. Each fibre was automatically picked up from the cassette and placed on the LEX 820 tensile testing machine. The equipment is a combination of an LDS0200 laser diffraction system for the diameter measurement and a tensile tester. The diameter of each fibre was measured for one axial and angular position. The reported value of the fibre diameter is the average of 250±10 measurements taken in the same fibre axial position. The tensile tests were carried out with a cross-head displacement rate of 0.6 mm/min. Once the cassette was placed in the tensile testing equipment, each test took about 1 min. The time taken by the automated equipment to test the single fibres was spent to mount more fibres onto another cassette and therefore optimize the process.

To remove the machine compliance from the calculations, 15–20 fibres for each fibre type were tested with a gauge length of 4 and 20 mm in addition to the gauge length of 12 mm used to obtain the large data sets. The displacement rate for these tests was 0.2 mm/min and 1 mm/min for the 4 and 20 mm gauge length, respectively. The machine compliance removal was performed using a method similar to the one described by ASTM C1557-14 [4]. By removing the system compliance, the stress-strain behaviour can be determined and hence the stiffness of the fibres calculated.

For more information on the materials and methods, please refer to [1].

## CRediT Author Statement

**Francisco Mesquita**: Conceptualization, Methodology, Software, Formal analysis, Investigation, Writing - original draft, Visualization; **Steve Bucknell**: Methodology, Software, Resources, Writing - review & editing; **Yann Leray**: Methodology, Resources, Writing - review & editing; **Stepan V. Lomov**: Conceptualization, Methodology, Writing - review & editing, Supervision; **Yentl Swolfs**: Conceptualization, Methodology, Formal analysis, Writing - review & editing, Supervision.

## Declaration of Competing Interest

The authors declare that they have no known competing financial interests or personal relationships that could have appeared to influence the work reported in this paper.
